# Lipschitz estimates for commutators of singular integral operators associated with the sections

**DOI:** 10.1186/s13660-017-1299-x

**Published:** 2017-01-25

**Authors:** Guangqing Wang, Jiang Zhou

**Affiliations:** College of Mathematics and System Sciences, Xinjiang University, Urumqi, 830046 Republic of China

**Keywords:** 42B20, 42B30, Hardy spaces, commutator, Lipschitz function, sections

## Abstract

Let *H* be Monge-Ampère singular integral operator, $b\in Lip_{\mathcal{F}}^{\beta}$, and $1/q=1/p-\beta$. It is proved that the commutator $[b,H]$ is bounded from $L^{p}(\mathbb{R}^{n},d\mu)$ to $L^{q}(\mathbb{R}^{n},d\mu)$ for $1< p<1/\beta$ and from $H^{p}_{\mathcal{F}}(\mathbb{R}^{n})$ to $L^{q}(\mathbb{R}^{n},d\mu)$ for $1/(1+\beta)< p\leq1$. For the extreme case $p=1/(1+\beta)$, a weak estimate is given.

## Introduction

In 1996, Caffarelli and Gutiérrez [1] introduced the new concept of a family of sections in studying real variable theory related to the Monge-Ampère equation. They defined the Hardy-Littlewood maximal operator $M_{\mathcal{F}}$ and $BMO_{\mathcal {F}}(\mathbb{R}^{n})$ spaces associated to sections, and the weak $(1,1)$ of $M_{\mathcal{F}}$ and the John-Nirenberg inequality for $BMO_{\mathcal{F}}(\mathbb{R}^{n})$ were obtained. Caffarelli and Gutiérrez [2] defined the singular integral operator *H* related to the Monge-Ampère equation and proved $L^{2}$-boundedness of it. Applying the theory of homogeneous spaces, Incognito [3] obtained a weak type $(1,1)$ estimate of *H*. In [4], Tang considered the commutator of Coifman-Rochberf-Weiss $[b,H]$ and obtained weighted estimates for the operator *H* and the commutator $[b,H]$, where $b\in BMO_{\mathcal{F}}$. And from [4], it follows that $[b,H]$ with $b\in BMO_{\mathcal{F}}$ is bounded on $L^{p}(\mathbb{R}^{n},d\mu)$ for $1< p<\infty$. Inspired by the above work, we will study the behaviors of commutator $[b,H]$ with $b\in Lip_{\mathcal{F}}^{\beta}$ acting on Lebesgue spaces $L^{p}(\mathbb{R}^{n},d\mu)$ and Hardy spaces $H^{p}_{\mathcal {F}}(\mathbb{R}^{n})$, where the Lipschitz spaces $Lip_{\mathcal {F}}^{\beta}$ and Hardy spaces $H^{p}_{\mathcal{F}}(\mathbb{R}^{n})$ associated with sections are defined by Lin [5].

As is well known, linear commutators are naturally appearing operators in harmonic analysis that have been extensively studied already. In general, the boundedness results of commutators in harmonic analysis can be used to characterize some important function spaces such as $BMO$ spaces, Lipschitz spaces, Besove spaces and so on (see [6–9]). Coifman *et al.* [10] applied the boundedness to some non-linear PDEs, which perfectly illustrate the intrinsic links between the theory of compensated compactness and the classical tools of harmonic and real analysis. As for some other essential applications to PDEs such as characterizing pseudodifferential operators, studying linear PDEs with measurable coefficients and the integrability theory of the Jacobians, interested researchers can refer to [11–14]. It is perhaps for this important reason that the boundedness of commutators attracted vast attention among researchers in harmonic analysis and PDEs. Thus, it is meaningful to identify the behaviors of commutator $[b,H]$ associated with the Monge-Ampère equation. In the sense of Euclidean space $\mathbb{R}^{n}$, the boundedness of commutator $[b,T]$ with $b\in Lip_{\beta}$ acting on Lebesgue spaces, is easily obtained by the inequality $|[b,T]f(x) |\leq CI_{\beta}(|f|)(x)$, where *T* is a Calderón-Zygmund singular integral operator and $I_{\beta}$ is the Riesz potential of order *β*. However, we cannot find a suitable operator to control the commutator $[b,H]$ with $b\in Lip_{\mathcal {F}}^{\beta}$ - just as controlling of $[b,T]$ with $b\in Lip_{\beta}$ - due to the particularity of the operator *H*. Therefore, in this paper we investigate $[b,H]$ directly, and obtain some relatively important properties.

This paper is organized as follows. In Section 2, we recall some elementary properties of sections. In the first part of Section 3, we demonstrate the $(L^{p},L^{q})$ boundedness of $[b,H]$ when $1< p< 1/\beta$ and $1/q=1/p-\beta$. It is worth mentioning that boundedness of $[b,H]$ from $L^{1}(\mathbb {R}^{n},d\mu)$ to weak $L^{1/(1-\beta)}(\mathbb{R}^{n},d\mu)$ is also obtained, which indicates the differences between the commutator $[b,H]$ with $b\in Lip_{\mathcal{F}}^{\beta}$ and that commutator with $b\in BMO_{\mathcal{F}}$. Based on these differences, in the second part of Section 3, we further discuss the behavior of the commutator $[b,H]$ acting on Hardy spaces $H^{p}_{\mathcal{F}}(\mathbb{R}^{n})$, and we see that the commutator $[b,H]$ is bounded from $H^{p}_{\mathcal{F}}(\mathbb{R}^{n})$ to $L^{q}(\mathbb{R}^{n},d\mu)$ if $1/(1+\beta)< p\leq1$ and $1/q=1/p-\beta $. For the extreme case $p=1/(1+\beta)$, we cannot get the $(H^{p},L^{q})$ boundedness of $[b,H]$, but we give a characterization. Instead of the boundedness in the extreme case, a weak estimate for $[b,H]$ is showed.

Now we recall the definition of sections which play an important role in the study of the Monge-Ampère equation and the linearized Monge-Ampère equation (see [1, 15–17]). For $x\in\mathbb{R}^{n}$ and $t>0$, let $S(x,t)$ denote an open and bounded convex subset of $\mathbb{R}^{n}$ containing *x*. The set $S(x,t)$ is called a *section* if the family $\mathcal{F}=\{ S(x,t)\subset\mathbb{R}^{n}: x\in\mathbb{R}^{n}\mbox{ and }t>0\}$ is monotone increasing in *t*, *i.e.*, $S(x,t)\subset S(x,t^{\prime})$ for $t\leq t^{\prime}$ which satisfies the following criteria:

(A) There exist constants $K_{1},K_{2},K_{3}$ and $\epsilon_{1},\epsilon _{2}$ such that given two sections $S(x_{0},t_{0})$, $S(x,t)$ with $t\leq t_{0}$ satisfying

$$S(x_{0},t_{0})\cap S(x,t)\neq\emptyset, $$

and given *T*, an affine transformation that “normalizes” $S(x_{0},t_{0})$, that is,

$$B(0,1/n)\subset T\bigl(S(x_{0},t_{0})\bigr)\subset B(0,1), $$

there exists $z\in B(0,K_{3})$ depending on $S(x_{0},t_{0})$ and $S(x,t)$ such that

1.1 $$ B(z,K_{2}(t/t_{0})^{\epsilon_{2}}\subset T\bigl(s(x,t) \bigr)\subset B\bigl(z,K_{1}(t/t_{0})^{\epsilon_{1}}\bigr) $$

and

$$T(z)\in B\bigl(z,(1/2)K_{2}(t/t_{0})^{\epsilon_{2}}\bigr). $$

Here $B(x,t)$ denotes the Euclidean ball centered at *x* with radius *t*.

(B) There exists a constant $\delta>0$ such that given a section $S(x,t)$ and $y\notin S(x,t)$, if *T* is an affine transformation that “normalizes” $S(x,t)$, then for any $0<\epsilon<1$

$$B\bigl(T(y),\epsilon^{\delta}\bigr)\cap T\bigl(S\bigl(x,(1-\epsilon)t \bigr)\bigr)=\emptyset. $$

(C) $\bigcap_{t>0}S(x,t)=\{x\}$ and $\bigcup_{t>0}S(x,t)=\mathbb{R}^{n}$.

In addition, we also assume that a Borel measure *μ* which is finite on compact sets is given, $\mu(\mathbb{R}^{n})=\infty$, and that it satisfies the *doubling property* with respect to $\mathcal {F}$, that is, there exists a constant *A* such that

1.2 $$ \mu\bigl(S(x,2t)\bigr)\leq A\mu\bigl(S(x,t)\bigr) $$

for any section $S(x,t)\in\mathcal{F}$. Throughout the paper, the letter *C* will denote a positive constant that may vary from line to line but remains independent of the main variables. We write $A\lesssim B$ to indicate that *A* is majorized by *B* times a constant independent of *A* and *B*, while the notation $A\thickapprox B$ denotes both $A\lesssim B$ and $B\lesssim A$. Finally, we denote $L^{p}_{\mu}:=L^{p}(\mathbb{R}^{n},d\mu)$ ($1\leq p\leq\infty$) simply.

## Elementary properties of section and notions

According to [18], the properties of (A) and (B) imply the following properties:

(D) There exists a constant $\theta\geq1$, depending only on $\delta , K_{1}$ and $\epsilon_{1}$, such that for any $y\in S(x,t)$,

2.1 $$ S(x,t)\subset S(y,\theta t) \quad\mbox{and}\quad S(y,t)\subset S(x,\theta t). $$

(E) There exists a quasi-metric $d(x,y)$ on $\mathbb{R}^{n}$ with respect to $\mathcal{F}$ defined by

$$d(x,y)=\inf\bigl\{ t:x\in S(y,t) \mbox{ and } y\in S(x,t)\bigr\} , $$

and its triangular constant is just the *θ* appearing in (D); that is,

2.2 $$ d(x,y)\leq\theta \bigl(d(x,z)+d(z,y) \bigr) \quad\mbox{for any } x,y,z\in \mathbb{R}^{n}. $$

Also,

2.3 $$ S(x,t/2\theta)\subset B_{d}(x,t)\subset S(x,t) \quad\mbox{for any } x \in \mathbb{R}^{n} \mbox{ and } t>0, $$

where $B_{d}(x,t)$ is a *d*-ball defined by $B_{d}(x,t):=\{y\in\mathbb {R}^{n}: d(x,y)< t\}$. Combining (1.2) and (2.3), one can see that there exists a constant $n_{0}>1$ and $2^{n_{0}}> A$ such that

2.4 $$ \mu\bigl(B_{d}(x,2r)\bigr)\leq2^{n_{0}}\mu \bigl(B_{d}(x,r)\bigr). $$

Thus, $(\mathbb{R}^{n},d,\mu)$ becomes a space of homogeneous type. Based on this, one can use the standard real analysis tools as the maximal function *Mf* and the sharp function $M^{\sharp}f$. In this paper, both of them are defined on $(\mathbb{R}^{n},d,\mu)$, namely

$$\begin{aligned}& Mf(x)=\sup_{r>0}\frac{1}{\mu(B(x,r))} \int_{B(x,r)}\bigl\vert f(y)\bigr\vert \,d\mu(y), \\& M^{\sharp}f(x)=\sup_{x\in B}\frac{1}{\mu(B)} \int _{B}\bigl\vert f(y)-f_{B}\bigr\vert \,d \mu(y) \approx\sup_{x\in B}\inf_{c} \frac{1}{\mu(B)} \int _{B}\bigl\vert f(y)-c\bigr\vert \,d\mu(y). \end{aligned}$$

Here and below, *B* is a *d*-ball and $f_{B}$ means the average of *f* on *B*. If we write $BMO(\mathbb{R}^{n}):=\{f: \|f\|_{BMO(\mathbb {R}^{n})}<\infty\}$ with $\|f\|_{BMO}=\|M^{\sharp}f\|_{L^{\infty}_{\mu }}$, then the $BMO_{\mathcal{F}}(\mathbb{R}^{n})$ space coincides with $BMO$ and $\|f\|_{BMO_{\mathcal{F}}(\mathbb{R}^{n})}\thickapprox\|f\| _{BMO(\mathbb{R}^{n})}$ (see [6]). Denote $M_{\delta}f(x)=M(|f|^{\delta })^{1/\delta}f(x)$ and $M_{\delta}^{\sharp}f(x)=M^{\sharp}(|f|^{\delta })^{1/\delta}(x)$.

Macías and Segovia [19] have found that the quasi-metric *d* can be replaced by another quasi-metric *ρ* such that $(\mathbb {R}^{n},\rho,\mu)$ is a normal space. Moreover, for the quasi-metric *ρ* there exist constants $C>0$ and $\epsilon\in(0,1)$ such that

2.5 $$ \textstyle\begin{cases} \rho(x,y)\approx\inf\{\mu(B_{d}):B_{d}\mbox{ are }d\mbox{-balls containing }x\mbox{ and }y\};\\ \mu(B_{\rho(x,r)})\approx r,\quad \forall x\in\mathbb {R}^{n}, r>0, \mbox{where } B_{\rho}(x,y):=\{y\in\mathbb{R}^{n}:\rho(x,y)< r\};\\ |\rho(x,y)-\rho(x',y)|\leq C\rho(x',y)^{\epsilon}[\rho(x,y)+\rho (x',y)]^{1-\epsilon}, \quad\forall x,x',y\in\mathbb{R}^{n}. \end{cases} $$

Let *ρ* satisfy (2.5) above and *f* be a continuous function on $\mathbb{R}^{n}$. Lin [5] defined Lipschitz spaces $Lip_{\mathcal {F}}^{\beta}$ associated with sections as follows.

### Definition 2.1

Let $0<\beta\leq1$. There exists a positive constant *C* such that

$$\sup_{\rho(x,y)\leq h}\bigl\vert f(x)-f(y)\bigr\vert \leq Ch^{\beta} $$

for $\forall h>0$. The “norm” of *f* in $Lip_{\mathcal{F}}^{\beta}$ is defined by the lower bound of the constants *C*.

Let *ϵ* be given in (2.5) above. Lin [5] found that the function spaces $\Lambda^{\beta}_{q,\mathcal{F}}$ and $Lip_{\mathcal {F}}^{\beta}$ coincide with equivalent norms for $0<\beta<\epsilon$ and $1\leq q\leq\infty$, where $\Lambda^{\beta}_{q,\mathcal{F}}$ denotes the Campanato spaces associated to the family $\mathcal{F}$ of the section. Also, he proved that $\Lambda^{\beta}_{q,\mathcal{F}}$ are the dual spaces of Hardy spaces $H^{p}_{\mathcal{F}}(\mathbb {R}^{n})$ ($1/2< p\leq1$).

For a locally integral function *b*, the commutator of Cofiman-Rochberg-Weiss $[b,H]$ is defined as follows:

$$[b,H]f(x)=b(x)Hf(x)-H(bf) (x)= \int_{\mathbb{R}^{n}} \bigl(b(x)-b(y) \bigr)k(x,y)f(y)\,d\mu(y), $$

where *H* is defined by the formula

$$Hf(x)= \int_{\mathbb{R}^{n}}k(x,y)f(y)\,d\mu(y), $$

with $k(x,y)=\sum_{i}k_{i}(x,y)$, and each kernel $k_{i}$ satisfies the following properties:

$$\begin{aligned}& \operatorname{supp}k_{i}(\cdot,y)\subset S_{i}(y), \quad\forall y\in \mathbb{R}^{n}; \qquad \operatorname{supp}k_{i}(x,\cdot)\subset S_{i}(x),\quad \forall x\in\mathbb{R}^{n}; \\& \int_{\mathbb{R}^{n}}k_{i}(x,y)\,d\mu(y)= \int_{\mathbb {R}^{n}}k_{i}(x,y)\,d\mu(x)=0, \quad\forall x,y\in \mathbb{R}^{n}; \\& \sup_{i} \int_{\mathbb{R}^{n}}\bigl\vert k_{i}(x,y)\bigr\vert \,d\mu (y)\leq C_{1}, \quad\forall x\in\mathbb{R}^{n}; \qquad\sup _{i} \int _{\mathbb{R}^{n}}\bigl\vert k_{i}(x,y)\bigr\vert \,d \mu(x)\leq C_{1},\quad \forall x\in\mathbb{R}^{n}; \end{aligned}$$

where $S_{i}(x)=S(x,2^{i})$ for any $x\in\mathbb{R}^{n},i\in\mathbb {Z}$. If *T* is an affine transformation that normalizes the section $S_{i}(y)$ then each $k_{i}$ satisfies the Lipschitz condition,

$$\bigl\vert k_{i}(u,y)-k_{i}(v,y)\bigr\vert \leq C_{2}\frac{1}{\mu(S_{i}(y))}|Tu-Tv|; $$

and, finally, if *T* is an affine transformation that normalizes the section $S_{i}(x)$ then $k_{i}$ satisfies the Lipschitz condition,

$$\bigl\vert k_{i}(x,u)-k_{i}(x,v)\bigr\vert \leq C_{2}\frac{1}{\mu(S_{i}(x))}|Tu-Tv|. $$

Caffarelli and Gutiérrez [2] obtained that *H* is bounded on $L^{2}_{\mu}$. Subsequently, Incognito [3] has given $L^{p}_{\mu}$ ($1< p<\infty$) and the weak-type $(1,1)$ estimate of *H*.

Using the property (D) and defining a function *σ* on $\mathbb {R}^{n}\times\mathbb{R}^{n}$ by $\sigma(x,y)=\inf\{t>0: y\in S(x,t)\}$, Incognito [3] obtained the following conclusions:

(E) $\sigma(x,y)\leq\theta\sigma(y,x)$ for all $x, y\in\mathbb{R}^{n}$.

(F) $\sigma(x,y)\leq\theta^{2}(\sigma(x,z)+\sigma(z,y))$ for all $x,y,z\in\mathbb{R}^{n}$.

It is easy to see that

2.6 $$ \sigma(x,y)< d(x,y)< \theta\sigma(x,y) \quad\mbox{for all } x,y\in \mathbb{R}^{n}, $$

and for a given section $S(x,t)$, $y\in S(x,t)$ if and only if $\sigma< t$.

## Main results

### Boundedness from $L^{p}_{\mu}$ to $L^{q}_{\mu}$

In this subsection, we discuss the property of the commutator acting on Lebesgue spaces.

#### Theorem 3.1

*Suppose that* $b\in Lip^{\beta}_{\mathcal{F}}$, $0<\beta< 1$. *If* $1/q=1/p-\beta$ *with* $1< p<1/\beta$, *then* $[b,H]$ *is bounded from* $L^{p}_{\mu}$ *to* $L^{q}_{\mu}$.

#### Theorem 3.2

*Suppose that* $b\in Lip^{\beta}_{\mathcal{F}}$, $0<\beta<\min\{1,\epsilon _{1}/n_{0}\}$, *where* $\epsilon_{1}$ *and* $n_{0}$ *are given in* (1.1) *and* (2.4) *respectively*. *Then the commutator* $[b,H]$ *is bounded from* $L^{1}_{\mu}$ *to weak* $L^{1/(1-\beta)}_{\mu}$.

In order to prove the theorems above, it is necessary to give the following lemmas.

#### Lemma 3.1

[20]

*Let* $0< p,\delta<\infty$ *and* $\omega\in A_{\infty}$. *There exists a positive* *C* *such that*

$$\int_{\mathbb{R}^{n}}M_{\delta}f(x)^{p}\omega(x)\,d\mu(x) \leq C \int _{\mathbb{R}^{n}} M^{\sharp}_{\delta}f(x)^{p} \omega(x)\,d\mu(x) $$

*for any smooth function* *f* *for which the left*-*hand side is finite*.

#### Lemma 3.2

[4]

*Let* $K(x,y)=\sum_{i}K_{i}(x,y)$. *Then there exists a constant* $C>0$ *such that*

$$\bigl\vert K(x,y_{0})-K(x,y)\bigr\vert +\bigl\vert K(y_{0},x)-K(y,x)\bigr\vert \leq C\frac{2^{-\epsilon_{1} k}}{\mu (S(y_{0},2^{k}\sigma(y_{0},x)) )} $$

*if* $\sigma(y_{0},x)\geq2^{k}4\theta^{2}\sigma(y_{0},y)$ *and* $k\geq0$.

#### Lemma 3.3

*Suppose that* $b\in Lip^{\beta}_{\mathcal {F}}$, *for* $0<\beta<1$. *Let* $0<\delta<1<r<1/\beta$. *Then there exists a constant* $C>0$ *such that*

$$\begin{aligned} M^{\sharp}_{\delta} \bigl([b,H]f \bigr) (x)\leq C\|b \|_{Lip^{\beta}_{\mathcal {F}}} \bigl(M_{\beta,r}(Hf) (x)+M_{\beta,r}(f) (x) \bigr) \end{aligned}$$

*for any smooth function* *f* *and every* $x\in\mathbb{R}^{n}$, *and where*

$$\begin{aligned} M_{\beta,r}(f) (x)=\sup_{x\in B} \biggl(\frac{1}{\mu(B)^{1-r\beta }} \int_{B}\bigl\vert f(y)\bigr\vert ^{r}\,d\mu(y) \biggr)^{1/r}. \end{aligned}$$

#### Proof

Observe that for any constant *λ*

$$[b,H]f(x)= \bigl(b(x)-\lambda \bigr)Hf(x)-H \bigl((b-\lambda)f \bigr) (x). $$

For any fixed ball $B=B(x,r)$. Decompose $f=f_{1}+f_{2}$, where $f_{1}=f\chi_{\bar{B}}$ with $\bar{B}=B(x,16\theta^{4}r)$. Let *λ* and $c_{B}$ be constants to be fixed in the proof. We write

$$\begin{aligned} & \biggl(\frac{1}{\mu(B)} \int_{B}\bigl\vert \bigl\vert [b,H]f(y)\bigr\vert ^{\delta }-|c_{B}|^{\delta}\bigr\vert \,d\mu(y) \biggr)^{1/\delta} \\ &\quad\leq \biggl(\frac{1}{\mu(B)} \int_{B}\bigl\vert \bigl(b(y)-\lambda \bigr)Hf(y) \bigr\vert ^{\delta}\,d\mu(y) \biggr)^{1/\delta} + \biggl(\frac{1}{\mu(B)} \int_{B}\bigl\vert H \bigl((b-\lambda)f_{1} \bigr) (y)\bigr\vert ^{\delta}\,d\mu(y) \biggr)^{1/\delta} \\ &\qquad{} + \biggl(\frac{1}{\mu(B)} \int_{B}\bigl\vert H \bigl((b-\lambda)f_{2} \bigr) (y)-c_{B}\bigr\vert ^{\delta}\,d\mu(y) \biggr)^{1/\delta} \\ &\quad:=L_{1}+L_{2}+L_{3}. \end{aligned}$$

For $L_{1}$, we fix $\lambda=b_{B}$. The Hölder inequality and (2.5) give us

$$\begin{aligned} L_{1} \lesssim\|b\|_{Lip^{\beta}_{\mathcal{F}}}\mu(B)^{\beta} \biggl( \frac {1}{\mu(B)} \int_{B}\bigl\vert Hf(y)\bigr\vert ^{r}\,d \mu(y) \biggr)^{1/r} \lesssim\|b\|_{Lip^{\beta}_{\mathcal{F}}}M_{\beta,r}(Hf) (x). \end{aligned}$$

From Kolmogorov’s inequality and (2.5), it follows that

$$\begin{aligned} L_{2}\lesssim\frac{\mu(B)^{1/\delta-1/r}}{\mu(B)^{1/\delta}} \biggl( \int _{B}\bigl\vert \bigl(b(y)-b_{B} \bigr)f_{1}(y)\bigr\vert ^{r}\,d\mu(y) \biggr)^{1/r} \lesssim\|b\|_{Lip^{\beta}_{\mathcal{F}}}M_{\beta,r}(f) (x). \end{aligned}$$

Finally, we take $c_{B}= (H ((b-b_{B})f_{2} ) )_{B}$. Then for any $x_{0}\in B$, Lemma 3.2, (2.5), (2.3), and (2.4) yield

$$\begin{aligned} L_{3} \leq&\frac{1}{\mu(B)} \int_{B}\bigl\vert H \bigl((b-\lambda)f_{2} \bigr) (y)- \bigl(H \bigl((b-b_{B})f_{2} \bigr) \bigr)_{B}\bigr\vert \,d\mu(y) \\ \leq&\frac{1}{\mu(B)^{2}} \int_{B} \int_{B} \int_{\mathbb {R}^{n}\backslash\bar{B}}\bigl\vert K(x,w)-K(z,w)\bigr\vert \bigl\vert b(w)-b_{B}\bigr\vert \bigl\vert f(w)\bigr\vert \,d\mu(w)\,d\mu (z)\,d\mu(y) \\ &{} \times\bigl\vert K(x,w)-K(z,w)\bigr\vert \bigl\vert f(w)\bigr\vert \,d\mu(w)\,d\mu(z)\,d\mu(y) \\ \lesssim&\|b\|_{Lip^{\beta}_{\mathcal{F}}} \sum_{k=1}^{\infty} \int _{2^{k}16\theta^{5}r< \sigma (x,w)\leq2^{k+1}16\theta^{5}r}\frac{\rho^{\beta}(x_{0},w)2^{-\epsilon_{1}k}}{ \mu (S(x,2^{k}16\theta^{5}r) )}\bigl\vert f(w)\bigr\vert \,d \mu(w) \\ \lesssim&\|b\|_{Lip^{\beta}_{\mathcal{F}}}M_{\beta,r}(f) (x) \sum _{k=1}^{\infty}2^{-\epsilon_{1}k} \\ \lesssim&\|b\|_{Lip^{\beta}_{\mathcal{F}}}M_{\beta,r}(f) (x). \end{aligned}$$

The estimates for $L_{1},L_{2}$, and $L_{3}$ indicate that the proof is completed. □

#### Lemma 3.4

[21]

*Let local integral function* $f\in L^{1}_{\mu}$ *and* $\alpha>0$. *Then there exists a family of balls* $\{B_{i}\}$ *such that*:

1. $|f(x)|\leq\alpha$, *for* *μ*-$a.e.x\in\mathbb{R}^{n}\backslash \bigcup_{i}B_{i}$;
2. $\frac{1}{\mu(B_{i})}\int_{B_{i}}|f(t)|\,d\mu(t)\leq C\alpha$;
3. $\sum^{\infty}_{i=1}\mu(B_{i})\leq\frac{C}{\alpha}\|f\| _{L^{1}_{\mu}}$;
4. *there exists an integer* $N\geq1$, *independent of* *f* *and* *λ*, *such that* $\sum_{i}\chi_{B_{i}}(x)\leq N$ *for* *μ*-*a*.*e*. $x\in \mathbb{R}^{n}$.

Now, with the lemmas above, we state the proof of our results.

#### Proof of Theorem 3.1

From Lemma 3.1 and Lemma 3.3 with $0<\delta<1<r<p$, it follows that

$$\begin{aligned} \bigl\Vert [b,H]f\bigr\Vert _{L^{q}_{\mu}} \leq&\bigl\Vert M_{\delta}\bigl([b,H]f\bigr)\bigr\Vert _{L^{q}_{\mu}} \\ \leq&\bigl\Vert M^{\sharp}_{\delta}\bigl([b,H]f\bigr)\bigr\Vert _{L^{q}_{\mu}} \\ \lesssim&\|b\|_{Lip^{\beta}_{\mathcal{F}}} \bigl(\bigl\| M_{\beta,r}(Hf)\bigr\| _{L^{q}_{\mu}}+ \bigl\Vert M_{\beta,r}(f)\bigr\Vert _{L^{q}_{\mu}} \bigr) \\ \lesssim&\|b\|_{Lip^{\beta}_{\mathcal{F}}}\|f\|_{L^{p}_{\mu}}. \end{aligned}$$

Thus, the proof of the theorem is completed. □

#### Proof of Theorem 3.2

For $f\in L^{1}_{\mu}$ and any $\alpha>0$, applying Lemma 3.4 with *α* replaced by $\alpha^{q_{0}}$ with $q_{0}=\frac{1}{1-\beta }$, we obtain, with the same notation as in Lemma 3.4, $f=g+h=g+\sum_{j}h_{j}$, where

$$g(x)=f(x)\chi_{\mathbb{R}^{n}\backslash\bigcup _{i}B_{i}}(x)+\sum_{j} (f \eta_{j} )_{B_{j}}\chi _{B_{j}}(x)\quad \mbox{and}\quad h_{j}(x)=f(x)\eta_{j}(x)- (f\eta_{j} )_{B_{j}}\chi_{B_{j}}(x) $$

with $\eta_{j}(x)=\frac{\chi_{B_{j}}(x)}{\sum_{j}\chi _{B_{j}}(x)}\chi_{\bigcup_{i}B_{i}}(x)$. By Lemma 3.4, it is easy to obtain the following properties:

(i) $|f(x)|\leq\alpha^{q_{0}}$, for *μ*-$a.e.x\in\mathbb {R}^{n}\backslash\bigcup_{j}B_{j}$;
(ii) $\frac{1}{\mu(B_{j})}\int_{B_{j}}|f(t)|\,d\mu(t)\leq C\alpha^{q_{0}}$;
(iii) $\sum^{\infty}_{j=1}\mu(B_{j})\leq\frac{C}{\alpha ^{q_{0}}}\|f\|_{L^{1}_{\mu}}$;
(iv) $\|g\|_{L^{1}_{\mu}}\leq C\|f\|_{L^{1}_{\mu}}$ and $|g(x)|\leq C\alpha^{q_{0}}$, for *μ*-a.e. $x\in\mathbb{R}^{n}$;
(v) each $h_{j}$ is supported in $B_{j}$, $\int_{\mathbb{R}^{n}}|h_{j}(x)|\,d\mu(x)\leq C\alpha^{q_{0}}\mu(B_{j})$ and $\int_{\mathbb{R}^{n}}h_{j}(x)\,d\mu(x)=0$.

Let $\bar{B}_{j}=B(z_{j},4\theta^{3}r_{j})$, we write

$$\begin{aligned} &\mu \bigl(x\in\mathbb{R}^{n}: \bigl\vert [b,H]f(x)\bigr\vert > \alpha \bigr) \\ &\quad\leq\mu \bigl(x\in\mathbb{R}^{n}: \bigl\vert [b,H]g(x)\bigr\vert >\alpha/2 \bigr) +\mu \biggl(x\in\bigcup_{j}\bar{B}_{j}: \bigl\vert [b,H]h(x)\bigr\vert >\alpha/2 \biggr) \\ &\qquad{} +\mu \biggl(x\in\mathbb{R}^{n}\backslash\bigcup_{j} \bar {B}_{j}: \bigl\vert [b,H]h(x)\bigr\vert >\alpha/2 \biggr) \\ &\quad:=K_{1}+K_{2}+K_{3}. \end{aligned}$$

Choose $1< p_{1}<1/\beta$ and $\frac{1}{q_{2}}=\frac{1}{p_{2}}-\beta$. The boundedness of $[b,H]$ from $L^{p_{2}}_{\mu}$ to $L^{q_{2}}_{\mu}$ and (iv) give us that

$$\begin{aligned} K_{1} \lesssim\alpha^{-q_{2}}\bigl\Vert [b,H]g\bigr\Vert ^{q_{2}}_{L^{q_{2}}_{\mu}} \lesssim\alpha^{-q_{2}}\|g \|^{q_{2}}_{L^{p_{2}}_{\mu}} \lesssim\alpha^{-q_{2}} \alpha^{q_{0}(p_{2}-1)\frac{q_{2}}{p_{2}}}\|f\| ^{\frac{q_{2}}{p_{2}}}_{L^{1}_{\mu}} \lesssim \alpha^{-q_{0}}\|f\|^{\frac{q_{2}}{p_{2}}}_{L^{1}_{\mu}}. \end{aligned}$$

By (iii), it is concluded that

$$K_{2}\leq\mu\biggl(\bigcup_{j}\bar{B}_{j}\biggr)\lesssim\sum _{j}\mu(B_{j}) \lesssim \alpha^{-q_{0}}\|f\|_{L^{1}_{\mu}}. $$

For $K_{3}$, we have

$$\begin{aligned} K_{3} \leq&\mu \biggl(x\in\mathbb{R}^{n}\big\backslash \bigcup_{j}\bar {B}_{j}: \sum_{j}\bigl|b(x)-b(z_{j})\bigr|\bigl|H(h_{j}) (x)\bigr|>\alpha/4 \biggr) \\ &{} +\mu \biggl(x\in\mathbb{R}^{n}\big\backslash \bigcup_{j} \bar{B}_{j}: \biggl|H \biggl(\sum_{j} \bigl(b-b(z_{j}) \bigr)h_{j} \biggr) (x)\biggr|>\alpha/4 \biggr) \\ :=&K_{31}+K_{32}. \end{aligned}$$

From (v), Lemma 3.2, (2.5), and (iii), it follows that

$$\begin{aligned} K_{31} \lesssim&\alpha^{-1}\sum _{j} \int_{\mathbb{R}^{n}\backslash \bigcup_{j}\bar{B}_{j}}\bigl\vert b(x)-b(z_{j})\bigr\vert \bigl\vert H(h_{j}) (x)\bigr\vert \,d\mu(x) \\ \lesssim&\alpha^{-1}\sum_{j} \int_{\mathbb{R}^{n}\backslash\bar {B}_{j}}\bigl|b(x)-b(z_{j})\bigr| \int_{B_{j}}\bigl\vert k(x,y)-k(x,z_{j})\bigr\vert \bigl\vert h_{j}(y)\bigr\vert \,d\mu(y)\,d\mu(x) \\ \lesssim&\|b\|_{Lip^{\beta}_{\mathcal{F}}}\alpha^{q_{0}-1} \biggl(\sum _{j}\mu(B_{j}) \biggr)^{\beta+1} \sum _{k=1}^{\infty}2^{(n_{0}\beta-\epsilon_{1})k} \\ \lesssim&\|b\|_{Lip^{\beta}_{\mathcal{F}}}\|f\|_{L^{1}_{\mu}}^{\beta +1} \alpha^{-q_{0}}. \end{aligned}$$

The boundedness of $[b,H]$ from $L^{1}_{\mu}$ to weak $L^{1}_{\mu}$, (2.5), (v), and (iii) give us that

$$\begin{aligned} K_{32} \lesssim&\alpha^{-1}\sum _{j} \int _{B_{j}}\bigl\vert b(x)-b(z_{j})\bigr\vert \bigl\vert h_{j}(x)\bigr\vert \,d\mu(x) \lesssim\|b \|_{Lip^{\beta}_{\mathcal{F}}}\alpha^{q_{0}-1} \biggl(\sum_{j} \mu(B_{j}) \biggr)^{\beta+1} \\ \lesssim&\|b\|_{Lip^{\beta}_{\mathcal{F}}}\|f\|_{L^{1}_{\mu}}^{\beta +1} \alpha^{-q_{0}}. \end{aligned}$$

Combining the estimates for $K_{1},K_{2}$ and $K_{3}$, one can finish the proof. □

### Boundedness from $H^{p}_{\mathcal{F}}(\mathbb{R}^{n})$ to $L^{q}_{\mu}$

In this subsection, we discuss the boundedness of the commutator $[b,H]$ on Hardy spaces $H^{p}_{\mathcal{F}}(\mathbb{R}^{n})$, and obtain the following results in which the symbols $\epsilon_{1}$ and $n_{0}$ are given in (1.1) and (2.4) respectively. Firstly, we recall the definition of the $(p,\infty)$-atoms and the atomic Hardy spaces $H^{p}_{\mathcal{F}}(\mathbb{R}^{n})$ with respect to a family $\mathcal{F}$ of sections and a doubling measure *μ*.

#### Definition 3.1

[5]

Let $1/2< p\leq1$. A function $a\in L^{\infty}_{\mu}$ is called a $(p,\infty)$-atom if there exists a section $S(x_{0},t_{0})\in\mathcal {F}$ such that

1. $\operatorname{supp}(a)\subset S(x_{0},t_{0})$;
2. $\int_{\mathbb{R}^{n}}a(x)\,d\mu(x)=0$;
3. $\|a\|_{L^{\infty}_{\mu}}\leq\mu(S(x_{0},t_{0}))^{-1/p}$.

The atomic Hardy space $H^{p}_{\mathcal{F}}(\mathbb{R}^{n})$ is defined by $H^{p}_{\mathcal{F}}(\mathbb{R}^{n})= \{f\in\mathcal{S}^{\prime}(\mathbb {R}^{n}): f(x)\stackrel{\mathcal{S}^{\prime}}{=}\sum_{j}\lambda_{j}a_{j}(x), \mbox{ }\mbox{each } a_{j}\mbox{ is a }(p,\infty)\mbox{-atom and }\sum_{j}|\lambda _{j}|^{p}<\infty \}$, where $\mathcal{S}(\mathbb{R}^{n})$ denotes the space of Schwartz functions and $\mathcal{S}^{\prime}(\mathbb{R}^{n})$ is the dual space of $\mathcal{S}(\mathbb{R}^{n})$. We define the $H^{p}_{\mathcal {F}}(\mathbb{R}^{n})$ norm of *f* by

$$\|f\|_{H^{p}_{\mathcal{F}}(\mathbb{R}^{n})}=\inf \biggl(\sum_{j}| \lambda_{j}|^{p} \biggr)^{1/p}, $$

where the infimum is taken over all decompositions of $f=\sum_{j}\lambda_{j}a_{j}$ above.

#### Theorem 3.3

*Suppose that* $b\in Lip^{\beta}_{\mathcal{F}}$, $0<\beta< \min\{ 1,\epsilon_{1}/n_{0}\}$. *If* $\frac{1}{1+\beta}< p\leq1$ *and* $1/q=1/p-\beta$, *then* $[b,H]$ *maps* $H^{p}_{\mathcal{F}}(\mathbb {R}^{n})$ *continuously into* $L^{q}_{\mu}$.

For the extreme case $p=1/(1+\beta)$, one gets the following characterization theorem.

#### Theorem 3.4

*Let* $b\in Lip^{\beta}_{\mathcal{F}}$, $0<\beta< \min\{1,\epsilon _{1}/n_{0}\}$. *Then the following statements are equivalent*.

- $[b,H]$ *raps* $H^{1/(1+\beta)}_{\mathcal{F}}$ *continuously into* $L^{1}_{\mu}$;
- *for any* $(1,\infty)$-*atom* *a* *supported in a section* $S=S(x_{0},r/2\theta)$ *and* $u\in S$,
  $$\biggl\vert \int_{\mathbb{R}^{n}}b(y)a(y)\,d\mu(y)\biggr\vert \int_{\bar{B}^{c}}\bigl|K(x,u)\bigr|\,d\mu (x)\lesssim1, $$
  *where* $\bar{B}=B(x_{0},16\theta^{4}r)$.

In general, the $(H^{p},L^{q})$ boundedness of $[b,H]$ fails for $p=1/(1+\beta)$, then we give a weak estimate instead.

#### Theorem 3.5

*Let* $b\in Lip^{\beta}_{\mathcal{F}}$, $0<\beta< \min\{1,\epsilon _{1}/n_{0}\}$. *Then* $[b,H]$ *maps* $H^{1/(1+\beta)}_{\mathcal{F}}(\mathbb {R}^{n})$ *continuously into weak* $L^{1}_{\mu}$.

Next, we show the proofs of the theorems above.

#### Proof of Theorem 3.3

Without loss of generality, we assume that $\|b\|_{Lip_{\mathcal{F}}^{\beta}}=1$. By Definition 3.1, we only need to prove that for any $(p,\infty)$-atom *a*, $\| [b,H]a\|_{L^{q}_{\mu}}\lesssim1$. Given a $(p,\infty)$-atom *a* with $\operatorname{supp}a\subset S=S(x_{0},r/2\theta)\in\mathcal{F}$. Let $B=B(x_{0},r), \bar{B}=B(x_{0},16\theta^{4}r)$. Then $S(x_{0},r/2\theta )\subset B(x_{0},r)$. Write

$$\bigl\Vert [b,H]a\bigr\Vert _{L^{q}_{\mu}}\leq \biggl( \int_{\bar{B}}\bigl\vert [b,H]a(x)\bigr\vert ^{q}\,d \mu (x) \biggr)^{1/q} + \biggl( \int_{\mathbb{R}^{n}\backslash\bar{B}}\bigl\vert [b,H]a(x)\bigr\vert ^{q}\,d \mu(x) \biggr)^{1/q} =I+II. $$

Choosing $1< p_{1}<1/\beta$ and $1/q_{1}=1/p_{1}-\beta$, and noting the $(p_{1},q_{1})$ boundedness of $[b,H]$ and the size condition of *a*, one can get

$$I\lesssim\bigl\Vert [b,H]a\bigr\Vert _{L^{q_{1}}_{\mu}}\mu(B)^{1/q-1/q_{1}} \lesssim \| a\|_{L^{p_{1}}_{\mu}}\mu(B)^{1/q-1/q_{1}}\lesssim\|a\|_{L^{\infty}_{\mu }} \mu(B)^{1/q+\beta}\lesssim1. $$

On the other hand, the cancellation condition of the atom *a* yields

$$\begin{aligned} II \leq& \biggl( \int_{\mathbb{R}^{n}\backslash\bar{B}}\biggl| \bigl(b(x)-b(x_{0}) \bigr) \int_{B} \bigl(K(x,y)-K(x,x_{0}) \bigr)a(y)\,d\mu (y)\biggr|^{q}\,d\mu(x) \biggr)^{1/q} \\ &{}+ \biggl( \int_{\mathbb{R}^{n}\backslash\bar{B}}\biggl| \int_{B}K(x,y) \bigl(b(x_{0})-b(y) \bigr)a(y)\,d \mu(y)\biggr|^{q}\,d\mu(x) \biggr)^{1/q} \\ :=&II_{1}+II_{2}. \end{aligned}$$

Lemma 3.2, (2.5), (2.3), and (2.4) imply that

$$\begin{aligned} II_{1} \lesssim&\frac{\|b\|_{Lip_{\mathcal{F}}^{\beta}}}{\mu(B)^{1/p}} \Biggl(\sum _{k=1}^{\infty} \int _{2^{k}16\theta^{5}r \leq\sigma(x,x_{0})< 2^{k+1}16\theta^{5}r}\biggl\vert \rho^{\beta}(x,x_{0})\\ &{}\times \int _{B}\bigl\vert K(x,y)-K(x,x_{0})\bigr\vert \,d\mu(y)\biggr\vert ^{q}\,d\mu(x) \Biggr)^{1/q} \\ \lesssim&\mu(B)^{1/q-1/p+\beta}\sum_{k=1}^{\infty}2^{k (n_{0}(\beta-1+1/q)-\epsilon_{1} )} \\ \lesssim&1. \end{aligned}$$

Finally, noting that

$$\bigl\Vert \bigl(b(x_{0})-b\bigr)a\bigr\Vert _{L^{q}_{\mu}} \lesssim\|b\|_{Lip_{\mathcal{F}}^{\beta }}\rho^{\beta}(x_{0},y)\|a \|_{L^{\infty}_{\mu}}\mu(B)^{1/q}\lesssim\mu (B)^{\beta} \mu(B)^{1/q-1/p}\lesssim1, $$

by the $(q,q)$ boundedness of *H*, one obtains

$$II_{2} = \biggl( \int_{\mathbb{R}^{n}\backslash\bar{B}}\bigl\vert H \bigl(\bigl(b(x_{0})-b \bigr)a \bigr) (x)\bigr\vert ^{q}\,d\mu(x) \biggr)^{1/q} \lesssim\bigl\Vert \bigl(b(x_{0})-b\bigr)a\bigr\Vert _{L^{q}_{\mu}}\lesssim1. $$

Combining the estimates for *I* and *II*, one can finish the proof. □

#### Proof of Theorem 3.4

Now that $[b,H]$ is bounded from $H^{1/(1+\beta)}_{\mathcal{F}}(\mathbb{R}^{n})$ to $L^{1}_{\mu}$ is equivalent to the fact that $\|[b,H]a\|_{L^{1}_{\mu}}\lesssim1$ holds for any $(1/(1+\beta),\infty)$ atom. Thus, we will study the behavior of $[b,H]$ acting on any $(1/(1+\beta),\infty)$ atom. Let *a* be an atom with $\operatorname{supp}\subset S=S(x_{0},r/2\theta)\in\mathcal{F}$, let $B=B(x_{0},r), \bar{B}=B(x_{0},16\theta^{4}r)$, then $S(x_{0},r/2\theta )\subset B(x_{0},r)$. For any $u\in S(x_{0},r/2\theta)$, one writes

$$\begin{aligned}{} [b,H]a(x) =&\chi_{B}(x)[b,H]a(x) +\chi_{\bar{B}^{c}}(x) \int_{B} \bigl(K(x,y)-K(x,u) \bigr) \bigl(b(y)-b(x_{0}) \bigr)a(y)\,d\mu(y) \\ &{} +\chi_{\bar{B}^{c}}(x) \bigl(b(x)-b(x_{0}) \bigr)Ha(x) + \chi_{\bar{B}^{c}}(x)K(x,u) \int_{B}b(y)a(y)\,d\mu(y) \\ :=& M_{1}+M_{2}-M_{3}-M_{4}. \end{aligned}$$

Similar to the estimate for *I* and $II_{1}$ in the proof of Theorem 3.3, we can show that $\|M_{1}\|_{L^{1}_{\mu}}\lesssim1$ and $\| M_{2}\|_{L^{1}_{\mu}}\lesssim1$. Using the vanishing condition, one can obtain

$$\begin{aligned} \|M_{3}\|_{L^{1}_{\mu}} \leq& \int_{\bar{B}^{c}}\bigl\vert b(x)-b(x_{0})\bigr\vert \int _{B}\bigl\vert K(x,y)-K(x,x_{0})\bigr\vert \bigl\vert a(y)\bigr\vert \,d\mu(y)\,d\mu(x) \\ \lesssim&\sum_{k=1}^{\infty}2^{k(n_{0}\beta-\epsilon_{1})} \\ \lesssim&1. \end{aligned}$$

The estimation above yields that $\|[b,H]a\|_{L^{1}_{\mu}}\lesssim1$ if and only if $\|M_{4}\|_{L^{1}_{\mu}}\lesssim1$. Hence the proof is finished. □

#### Proof of Theorem 3.5

Let $f\in H^{1/(1+\beta)}_{\mathcal {F}}(\mathbb{R}^{n})$ and $f(x)=\sum_{i}\lambda_{i}a_{i}(x)$ with each $a_{i}$ an $(1/(1+\beta),\infty)$-atom and $\sum_{i}|\lambda_{i}|^{1/(1+\beta)}<\infty$. Suppose that $\operatorname{supp}a\subset S_{i}=S(x_{i},r_{i})$. Write

$$\begin{aligned}{} [b,H]f(x) =&\sum_{i}\lambda_{i} \bigl(b(x)-b(x_{i}) \bigr)Ha_{i}(x)\chi_{\bar{B}}(x) + \sum_{i}\lambda_{i} \bigl(b(x)-b(x_{i}) \bigr)Ha_{i}(x)\chi_{(\bar {B})^{c}}(x) \\ &{} -H \biggl(\sum_{i}\lambda_{i} \bigl(b-b(x_{i}) \bigr)a_{i} \biggr) (x) :=J_{1}+J_{2}+J_{3}. \end{aligned}$$

By the Hölder inequality and the $(2,2)$ boundedness of *H*, we have

$$\bigl\Vert \bigl(b-b(x_{i}) \bigr) (Ha_{i}) \chi_{\bar{B}}\bigr\Vert _{L^{1}_{\mu}}\lesssim1. $$

Using the same method as $M_{3}$ together with $0<\beta<\epsilon _{1}/n_{0}$, it is easy to check

$$\begin{aligned} \bigl\Vert \bigl(\bigl(b-b(x_{i})\bigr) (Ha_{i}) \chi_{\bar{B}^{c}} \bigr)\bigr\Vert _{L^{1}_{\mu }}\lesssim1. \end{aligned}$$

So, we have

3.1 $$ \mu\bigl(\bigl\{ x\in\mathbb{R}^{n}: |J_{j}|>\lambda/3\bigr\} \bigr)\lesssim\lambda^{-1}\| J_{j}\|_{L^{1}_{\mu}}\lesssim \lambda^{-1}\sum_{i}|\lambda _{i}|,\quad j=1,2. $$

Finally, noting that

$$\bigl\Vert \bigl(b-b(x_{i})\bigr)a\bigr\Vert _{L^{1}_{\mu}} \lesssim\|b\|_{Lip_{\mathcal{F}}^{\beta }}\rho^{\beta}(x_{i},y)\|a \|_{L^{\infty}_{\mu}}\mu(B)\lesssim\mu (B)^{\beta}\mu(B)^{1-(1+\beta)} \lesssim1, $$

and we have the weak $(1,1)$ boundedness of *H*, one obtains

3.2 $$ \mu\bigl(\bigl\{ x\in\mathbb{R}^{n}: |J_{3}|>\lambda/3\bigr\} \bigr)\lesssim\biggl\| \sum_{i}\lambda_{i} \bigl(b-b(x_{i}) \bigr)a_{i}\biggr\| _{L^{1}_{\mu}} \lesssim \lambda^{-1}\sum_{i}|\lambda_{i}|. $$

Equations (3.1) and (3.2) imply that the proof is completed. □

## Conclusions

The authors prove the commutator $[b,H]$ is bounded from $L^{p}(\mathbb {R}^{n},d\mu)$ to $L^{q}(\mathbb{R}^{n},d\mu)$ for $1< p<1/\beta$ and from $H^{p}_{\mathcal{F}}(\mathbb{R}^{n})$ to $L^{q}(\mathbb{R}^{n},d\mu )$ for $1/(1+\beta)< p\leq1$ and give the weak estimate at the extreme case $p=1/(1+\beta)$ as well, which may give us an essential tool to study the linear or non-linear Monge-Ampère equation. It is a pity that we do not characterize the Lipschitz spaces $Lip^{\beta}_{\mathcal {F}}$ with the boundedness of it due to the particularity of the operator *H*. But in order to provide more useful ways to study the equation we will continue to perform this work in the future. Moreover, the smoothing effect and the compactness of the commutator $[b,H]$ can be investigated as well.
